# Recovery From Eccentric Squat Exercise in Resistance-Trained Young and Master Athletes With Similar Maximum Strength: Combining Cold Water Immersion and Compression

**DOI:** 10.3389/fphys.2021.665204

**Published:** 2021-09-10

**Authors:** Julian Schmidt, Alexander Ferrauti, Michael Kellmann, Florian Beaudouin, Mark Pfeiffer, Nicola Reiner Volk, Jan Martin Wambach, Oliver Bruder, Thimo Wiewelhove

**Affiliations:** ^1^Faculty of Sport Science, Ruhr University Bochum, Bochum, Germany; ^2^School of Human Movement and Nutrition Sciences, The University of Queensland, Brisbane, QLD, Australia; ^3^Institue of Sports and Preventive Medicine, Saarland University, Saarbrücken, Germany; ^4^Institute of Sports Science, Johannes-Gutenberg University, Mainz, Germany; ^5^Faculty of Medicine, Ruhr University Bochum, Bochum, Germany; ^6^Contilia Heart and Vascular Center, Elisabeth-Hospital, Essen, Germany; ^7^IST University of Applied Sciences, Düsseldorf, Germany

**Keywords:** aging, mixed-method recovery intervention, crossover, resistance training, muscle fatigue, muscle soreness

## Abstract

The aim of this study was to investigate whether recovery from eccentric squat exercise varies depending on age and to assess whether the use of a mixed-method recovery (MMR) consisting of cold water immersion and compression tights benefits recovery. Sixteen healthy and resistance-trained young (age, 22.1±2.1years; *N*=8) and master male athletes (age, 52.4±3.5years; *N*=8), who had a similar half squat 1-repetition maximum relative to body weight, completed two identical squat exercise training sessions, separated by a 2-week washout period. Training sessions were followed by either MMR or passive recovery (PR). Internal training loads [heart rate and blood lactate concentration (BLa)] were recorded during and after squat sessions. Furthermore, maximal voluntary isometric contraction (MVIC) force, countermovement jump (CMJ) height, resting twitch force of the knee extensors, serum concentration of creatine kinase (CK), muscle soreness (MS), and perceived physical performance capability (PPC) were determined before and after training as well as after 24, 48, and 72h of recovery. A three-way mixed ANOVA revealed a significant time effect of the squat protocol on markers of fatigue and recovery (*p*<0.05; decreased MVIC, CMJ, twitch force, and PPC; increased CK and MS). Age-related differences were found for BLa, MS, and PPC (higher post-exercise fatigue in younger athletes). A significant two-way interaction between recovery strategy and time of measurement was found for MS and PPC (*p*<0.05; faster recovery after MMR). In three participants (two young and one master athlete), the individual results revealed a consistently positive response to MMR. In conclusion, master athletes neither reach higher fatigue levels nor recover more slowly than the younger athletes. Furthermore, the results indicate that MMR after resistance exercise does not contribute to a faster recovery of physical performance, neuromuscular function, or muscle damage, but promotes recovery of perceptual measures regardless of age.

## Introduction

It has been discussed that recovery processes during the days after intensive physical exercise are modified in elderly athletes (master athletes) compared to the age of peak performance (Fell and Williams, 2008). Master athletes experience either greater muscle damage, slower repair, or a combination of both effects, which can be attributed to physiological and social lifestyle changes beginning in the fourth decade of life effects (Smith and Norris, 2002; Fell and Williams, 2008). A deeper insight into the specificity of fatigue and recovery patterns of younger and older athletes during the days after an intensive bout of exercise is of practical interest for an appropriate training prescription.

Regarding the physiological processes of aging, a reduction in muscle cross-sectional area and protein synthesis rate, a loss in type II muscle fibers and motor units, and a change in muscle contractile properties and stiffness have been shown (Feldman et al., 2002; Sinha-Hikim et al., 2002; Lavender and Nosaka, 2006; Brunner et al., 2007; Fell and Williams, 2008; Borges et al., 2016; McCormick and Vasilaki, 2018). Anabolic changes are mainly attributed to a 1–3% decline per year in circulating testosterone concentrations (1.6% in total and 2–3% in bioavailable testosterone) and a reduced testosterone response to resistance exercise beyond 35–40years (Vingren et al., 2010). In addition to the physiological changes, increasing sedentary lifestyles coming along with a decrease in training volume and intensity appear to play a reinforcing effect in any observed decrements in performance in older athletes (Hawkins et al., 2003; Foster et al., 2007; Borges et al., 2016).

An age-related decrease in performance might impact the recovery process (Borges et al., 2016). Therefore, some studies compared the recovery kinetics between younger and older athletes after a standardized training load, but most of them have been conducted in older and sedentary participants (Fell et al., 2008; Bieuzen et al., 2010; Easthope et al., 2010; Borges et al., 2016). This approach seems to be inappropriate since age-related changes might be overlaid by differences in training status of sedentary participants. Consequently, more research is needed regarding the effects of aging on muscle damage and recovery needs in tightly controlled populations of younger and older athletes with comparable performance and training status (Fell and Williams, 2008).

In practice, various post-exercise recovery strategies, like active recovery (Wiewelhove et al., 2021), massage (Poppendieck et al., 2016), foam rolling (Wiewelhove et al., 2019), compression clothes (Born et al., 2013), and cooling interventions (Poppendieck et al., 2013), are used to accelerate recovery after intense physical exercise. While most of these interventions are still lacking sufficient evidence, cold water immersion (CWI) has been shown to have at least small effects on performance restoration (Poppendieck et al., 2013). This is attributed to several mechanisms, including muscle temperature decrease, analgesic effects, peripheral vasoconstriction, and reduction of edema as well as a reduction of cellular metabolism, which may reduce inflammation and muscle damage (Wilcock et al., 2006). Regarding their physiological effects, CWI and compression garments are likely to be consistently helpful during muscle recovery and repair following intensive strength training (Ibegbuna et al., 2003; Davies et al., 2009; Leeder et al., 2012; Born et al., 2013; Poppendieck et al., 2013; Zinner et al., 2017). So far, no studies have compared the recovery intervention effects among different age groups.

The present study aims at expending the current state of knowledge, since two aspects are included which have not been sufficiently considered in exercise research with aging athletes so far. First, the selection of participants from different age groups was tightly matched regarding their training status and primary strength performance markers. Second, only few studies compared the recovery intervention effects after intensive strength training in individuals of different age groups in a controlled and ecologically valid cross-over design. Therefore, the purposes of this study were to compare the time course of fatigue and recovery from eccentric squat exercise and the effects of a mixed-method recovery (MMR) intervention in resistance-trained young and older individuals of similar performance. Considering the above-mentioned age-related anabolic differences, our hypotheses were that (1) older individuals recover slower and (2) might benefit more from additional external applied recovery aids after exercise-induced muscle fatigue.

## Materials and Methods

### Participants

Sixteen healthy and resistance-trained recreational male athletes (regularly competing at regional or national levels in a team or individual sport modality), drawn from two age groups, eight young athletes (age, 22.1±2.1years) and eight master athletes (age, 52.4±3.5years), took part in this study. Athletes had to meet the following inclusion criteria: a minimal half squat 1-repetition maximum (1RM) of at least 120% of the individual body weight and a minimum of two resistance training sessions per week for at least the past year. The baseline physical and performance characteristics of the athletes are shown in Table 1. After being informed about the exercise protocols and all possible risks associated with participation in the investigation, the participants provided their written consent to participate in all procedures. Normal ECG and the absence of cardiovascular, pulmonary, and orthopedic diseases were confirmed in a preliminary health examination. Ethical approval was received from the local ethics committee.

**Table 1:** Baseline physical characteristics of the participants.

Measure	Young athletes (*n*=8)	Older athletes (*n*=8)	Differences between age groups	
	Mean± *SD*		*p*	ES
Age (years)	22.1 ± 2.1	52.4 ± 3.5	0.001	10.85
Height (cm)	179.9 ± 5.2	177.8 ± 7.8	0.519	0.33
Weight (kg)	79.8 ± 8.0	78.9 ± 8.5	0.832	0.11
BMI (kg/m^2^)	24.6 ± 2.1	25.0 ± 2.5	0.742	0.17
Fat (%)	12.8 ± 3.2	15.2 ± 4.2	0.228	0.63
Overall muscle mass (kg)	39.8 ± 3.4	36.7 ± 4.1	0.115	0.83
Leg muscle mass (kg)	20.7 ± 1.8	19.5 ± 2.6	0.273	0.57
Core muscle mass (kg)	30.9 ± 2.7	28.7 ± 3.2	0.141	0.72
1RM_est_ (kg)	112.0 ± 13.9	105.8 ± 16.8	0.431	0.41
1RM_est_ (%)	141.1 ± 14.8	135.0 ± 22.8	0.525	0.32
Testosterone (pg/ml)	17.4 ± 5.0	8.3 ± 1.3	0.001	2.50

*BMI, body mass index; 1RM_est_, estimated one repetition maximum; and ES, effect size*.

### Experimental Design

**Figure 1:**
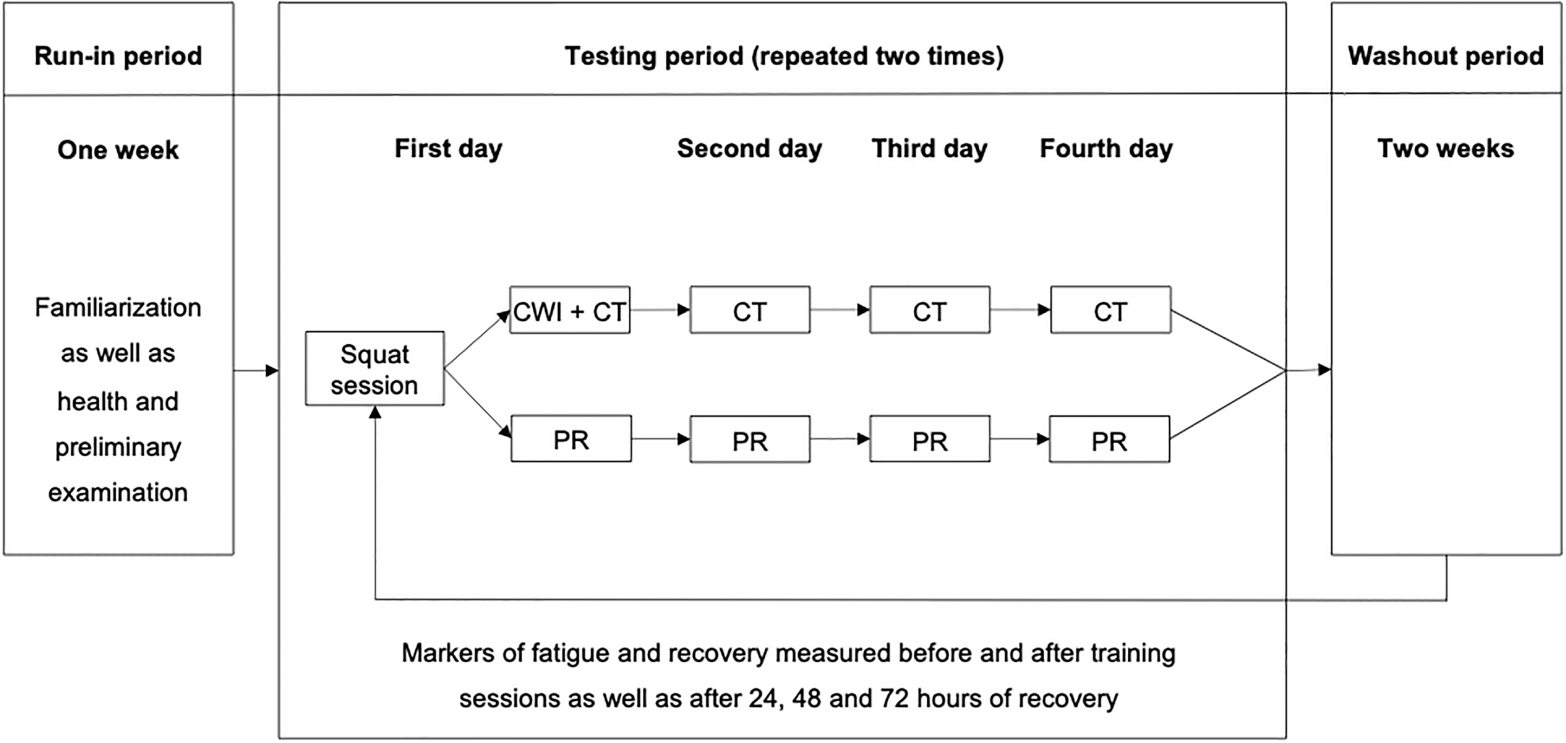
Experimental design. CWI, cold water immersion; CT, compression tights; and PR, passive recovery.

After an initial preliminary health examination and familiarization with the performance tests, the subjective measures, and the exercise protocol, all athletes completed two fatiguing squat exercise protocols, separated by a 2-week washout period. The two sessions were followed by either an MMR intervention (consisting of CWI and compression tights) or passive recovery (PR, i.e., the participants did not use any putative recovery modalities throughout the testing period). For the assignment to one of two groups, athletes were matched according to their age. Then, within each group, the participants were randomly assigned to one of two subgroups. The first subgroup performed MMR after the first exercise protocol, whereas the other subgroup used PR. After the second training protocol, recovery conditions were interchanged. Maximal voluntary isometric contraction (MVIC) force in the half squat and seated leg press, resting twitch force of the knee extensors, countermovement jump (CMJ) height, serum concentration of creatine kinase (CK), muscle soreness, and perceived physical performance capability (PPC) were determined pre- and post-training (post-0) as well as after 24h (post-24), 48h (post-48), and 72h (post-72) of recovery (Figure 1). These parameters were measured as they were shown to represent changes in fatigue and recovery after strength training with adequate sensitivity (Raeder et al., 2016a).

The athletes were instructed to consume a carbohydrate-rich meal two hours before and after all testing and training sessions, to arrive at the sessions in a hydrated state, and to avoid strenuous exercise for 96h before the training sessions. During the 72h recovery after the intensive squat exercise protocols, the participants were prohibited from any additional training. The participants were also instructed to avoid using any additional putative recovery modalities throughout the testing periods. Each athlete was examined at approximately the same time of day to reduce the effects of diurnal variation. The tests were always conducted in the same facility and in the same order: assessment of current muscle soreness and PPC, capillary blood collection, MVIC force in the half squat and leg press, resting twitch force, and CMJ performance. To minimize possible learning effects, the participants were thoroughly familiarized with the MVIC and CMJ tests during the run-in period. The participants were also asked to maintain their normal dietary intake, to refrain from nutritional supplements, caffeine, and alcohol intake during the entire study, to use their normal social and sleep rhythms, and to avoid using additional putative recovery modalities. The participants were verbally questioned regularly to ensure they adhered to the rules and requirements and their feedback suggested they followed the rules. In addition, all athletes were required to record dietary intake during the first testing periods using the Freiburger Nutrition Protocol (Nutri-Science GmbH, Freiburg, Germany). Energy intake and macronutrient distribution were analyzed using the MyFitnessPal Web site.^1^ After the first testing period, the participants were instructed to replicate their dietary intake throughout the second testing period. Average daily energy intake and macronutrient distribution during the first testing period was as follows: young athletes, 2,678±513kcal, 19±3% fat, 58±4% carbohydrates, and 23±5% protein; master athletes, 2,415±839kcal, 18±5% fat, 63±10% carbohydrates, and 19±6% protein.

### Exercise Protocol

A standardized squat exercise protocol was designed to induce a temporary state of fatigue while remaining tolerable for the athletes (i.e., the participants were able to maintain proper exercise technique, intensity, and volume throughout the squat exercise protocol). The squat exercise was selected as the basic movement because of its similar biomechanical and neuromuscular characteristics compared to many athletic movement activities. Furthermore, squat exercises are widely used in resistance training regimens (Schoenfeld, 2010; Raeder et al., 2016b). A Smith machine with a guided barbell (Technogym, Cesena, Italy) was used for training. The participants performed half squats (i.e., 90° knee angle) using safety stoppers to standardize the range of motion. The exercise protocol contained 9 sets of 8 repetitions and a final loading set performed to momentary muscular failure at an intensity of 70% of the participant’s individual 1RM, with a cadence per repetition of 4s in eccentric mode and 2s in concentric mode, approximately 480s of total time under tension for all 10 sets, and 3min rest intervals between sets (Raeder et al., 2016b).

The training sessions were preceded by a 10-min standardized dynamic warm-up protocol (jogging, high knee skipping and running, heel ups, lunges with trunk rotation, straight-leg skipping, straight-leg deadlift walk, deep squatting, and bilateral jumps), core stability exercises (plank and side plank), and five repetitions of the athlete’s 50 and 70% 1RM in a half squat. All sessions were supervised by the same research fellows, and the number of repetitions in the final sets was registered. Additionally, heart rates (HRs) were monitored and recorded at 1s intervals during each training session (V800, Polar Electro, Kempele, Finland), blood lactate concentrations (BLa) were measured after the last set of half squats, and the work intensity was rated by the participants immediately after the last set using a 10-point category-ratio rating of perceived exertion (RPE) scale (Alexiou and Coutts, 2008). Mean and peak HRs, BLa concentrations, and RPE values for the individual exercise sessions were used for later analysis.

### Recovery Intervention

After the exercise sessions, participants performed the recovery (i.e., MMR) or control condition (i.e., PR). MMR consisted of whole-body CWI immediately followed by wearing full-length compression tights. CWI was applied continuously for 15min, during which the participants remained seated in tubs while immersing their entire bodies, excluding their heads and necks. The water temperature was maintained at 12±1°C by adding crushed ice as needed and controlled by the use of a liquid-in-glass thermometer. This protocol was adopted because a recent review demonstrated the presence of a dose–response relationship with CWI, indicating that a water temperature of 11–15°C and an immersion time of 11–15min could provide the most beneficial effect on muscle soreness (Machado et al., 2016). The participants were also instructed to perform circular motions with their legs every 2min to prevent the formation of a warmer boundary layer surrounding the skin (Wiewelhove et al., 2018). Full-length compression tights with distal-to-proximal pressure gradient (medi GmbH & Co. KG, Bayreuth, Germany) were individually fit to participants based on upper and lower leg circumferences (mean pressure ranged from 18 to 21 mmHg). These were made of 75% polyamide and 20% elastane and worn for 48h after CWI. This application time was chosen based on the previous published results showing that compression clothing achieves the most beneficial effects 12 to 24h after exercise, if they are used for recovery purposes (Born et al., 2013). Compression tights were not worn while showering as well as during post-24, post-48, or post-72 measurements.

### Measurements

During the preliminary examination, height, weight, and body composition were recorded with a standard stadiometer and a biometrical impedance analysis system (InBody Deutschland, Eschborn, Germany), respectively. Additionally, maximal dynamic strength (estimated 1RM, 1RM_est_) in the half squat with a cadence per repetition of 4s in eccentric mode and 2s in concentric mode was determined using a Smith machine with a guided barbell (Technogym, Cesena, Italy), following a previously described standardized protocol (Raeder et al., 2016b). 1RM_est_ was used to calculate the athletes’ individual training loads in half squat exercises throughout the testing periods.

Maximal voluntary isometric contraction force in the half squat and the leg press was measured by recording the maximal isometric force output in Newton (N), using a Smith machine with a guided barbell (Technogym) and a seated leg press machine (proxomed Medizintechnik GmbH, Alzenau, Germany), respectively. For the MVIC half squat test, the participants were directed to position themselves under the barbell into a shoulder bride stand with an external foot rotation of 5–10°. Perpendicular to the middle of the barbell, a floor marker functioned as a point of reference to stand parallel and to standardize the adjustment of the axis of the ankle joint. Subsequently, the bar was lowered and mechanically locked into a testing position corresponding to a knee joint angle of 90° using a customary goniometer. This testing position was previously determined for each participant and kept constant throughout the study. The MVIC force in the seated leg press was tested with hip and knee flexion angles of 130° and 90°, respectively. For each of the two MVIC tests, the athletes performed three maximum contractions of 3s duration, which were separated by 2min of rest. The MVIC force of each attempt was measured by a calibrated strain gauge (ME-Meßsysteme GmbH, Hennigsdorf, Germany) and sampled using an analog-to-digital converter (ME-Meßsysteme) and customized computer software. The MVICs with the highest force for each of the two tests were used for later analysis.

Muscle contractility of the knee extensors of the left leg was assessed from the electrically evoked resting twitch force with the athletes seated in the leg press machine (proxomed Medizintechnik). Electrical stimuli to the knee extensors were delivered *via* a cathode (8×13cm) and a large anode (10×18cm) placed over the proximal and distal quadriceps femoris, respectively. Before electrode placement, the skin was shaved, lightly abraded, and washed to remove surface layers of dead skin, hair, and oil. The position of the electrodes was marked with indelible ink to insure that they were placed in the same location during subsequent trials (Souron et al., 2018). A fixed current of 110mA was delivered using a stimulator (TMG-BMC, Ljubljana, Slovenia). Three single twitch forces on the relaxed state were determined, and the mean twitch force was used for later analysis.

The CMJs were performed on a contact platform (Haynl-Elektronik GmbH, Schönebeck, Germany) with the hands placed on the hips. When performing the CMJs, the athletes dropped down to a self-selected level before jumping to the maximum height. Flight time was used to calculate jump height. Athletes performed three maximal CMJs, and the maximum jump height was used for later analysis.

Capillary whole-blood samples were taken from the hyperemized earlobe and analyzed for BLa and CK. BLa samples were taken with 20-μl capillaries, hemolyzed in 1-ml micro-test tubes, and underwent amperometric-enzymatic analysis using the Biosen S-Line Sport (EKF-diagnostic GmbH, Barleben, Germany). CK samples were collected with a 200-μl capillary blood collection system prepared for serum collection with a coagulation inducer and a separating gel (KABE Labortechnik GmbH, Nümbrecht-Elsenroth, Germany). This was positioned upright to clot at room temperature for 10min, subsequently centrifuged, and then analyzed by the COBAS INTEGRA 400 plus (Roche Diagnostics GmbH, Mannheim, Germany).

Muscle soreness was recorded using the 11-point Numerical Pain Rating Scale (NPRS). The NPRS includes a horizontal bar marked with whole numbers from 0 to 10 and is anchored with the phrases “No Pain” on the left and “Worst Imaginable Pain” on the right (Hawker et al., 2011). The athletes were asked to palpate their lower limb muscles and then to indicate the NPRS number that best described their general amount of muscle pain at the time of the measurement. Perceived PPC was determined using the Short Recovery and Stress Scale (SRSS), which consists of a Short Recovery Scale and a Short Stress Scale with four items each on a scale ranging from 0 (does not apply at all) to 6 (fully applies; Kellmann and Kölling, 2019). In this study, only the PPC item was used. The internal consistency for the Short Recovery Scale and the Short Stress Scale was deemed acceptable (Cronbach’s Alpha, 0.72 and 0.75, respectively; Kellmann and Kölling, 2020), and previous research indicated the SRSS’s sensitivity to exercise-induced fatigue (Hitzschke et al., 2017).

### Statistical Analysis

Statistical analysis was conducted with Microsoft Excel ver. 16.30 (Microsoft Corporation, Redmond, WA, United States) and IBM SPSS Statistics ver. 27 (IBM Corporation, Armonk, NY, United States). All data were expressed as mean, standard deviation (SD), and 95% confidence intervals (95% CI). Normal distribution was assessed by Shapiro–Wilk test. In cases of non-normal distribution, data were log-transformed prior to statistical analysis to improve normality and variance homogeneity.

First, values for MVIC force, resting twitch force, CMJ height, serum concentration of CK, muscle soreness, and PPC were analyzed using a three-way mixed ANOVA employing the two within-subject factors recovery strategy (two levels: MMR and PR) and time of measurement (five levels: pre- and post-training and after 24, 48, and 72h of recovery) and the between-subject factor age group (two levels: young and master athletes). If Mauchly’s test of sphericity indicated that the assumption of sphericity was violated, the results of the mixed ANOVA were interpreted using the Greenhouse–Geisser correction. Statistical significance was set at *p*<0.05.

Second, the magnitude of the individual differences in the post-training to post-48-h changes in markers of fatigue between MMR and PR was quantified using the effect size. Individual effect sizes were determined by calculating the individual differences in the changes between MMR and PR and dividing the result by the pooled individual pretraining SD. To represent the decision-making process common in practice (Buchheit et al., 2020), a substantial difference in the changes between MMR and PR was accepted when the effect size was greater than 0.35 (Rhea, 2004; Turner et al., 2015).

Third, training loads were analyzed using a two-way mixed ANOVA with the within-subject factor squat exercise session (two levels: exercise session followed by MMR and exercise session followed by PR) and the between-subject factor age group (two levels: young and master athletes).

## Results

**Figure 2:**
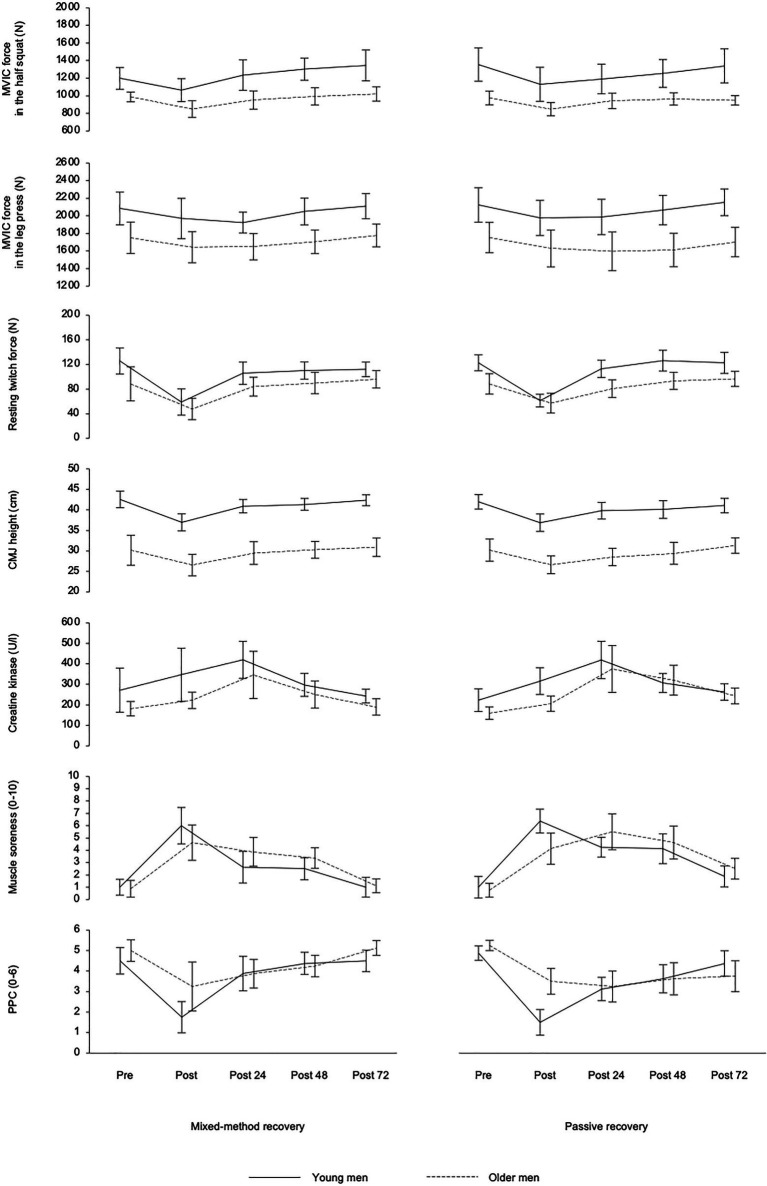
Mean (± 95% confidence intervals) of the young and older athletes in the two recovery conditions for maximal voluntary isometric contraction (MVIC) force in the half squat and leg press, resting twitch force of the knee extensors, countermovement jump (CMJ) height, serum concentration of creatine kinase, muscle soreness, and perceived physical performance capability (PPC) before (Pre) and after (Post) squat exercise sessions as well as after 24h (post-24), 48h (post-48), and 72h (post-72) of recovery.

The three-way mixed ANOVA detected a statistically significant fatigue-related within-subject effect of time of measurement for all markers of fatigue and recovery (*p*<0.05; Table 2), as MVIC force, resting twitch force, CMJ height, serum concentration of CK, muscle soreness, and PPC declined or increased and returned to baseline levels after 72h of recovery (Figure 2). In addition, a statistically significant between-subject effect of age group was found for MVIC force and CMJ height (*p*<0.05), as they were higher in young athletes as compared with master athletes. A statistically significant two-way interaction between recovery strategy and time of measurement was found for muscle soreness and PPC (*p*<0.05), as they recovered faster between post-0 and post-72 for MMR as compared with PR. Furthermore, there was a statistically significant two-way interaction between time of measurement and age group for perceptual fatigue measures (*p*<0.05), as muscle soreness was higher and PPC was lower at post0 in young athletes as compared with master athletes. However, at post-24, post-48, and post-72, muscle soreness and PPC were not different between age groups. There was no statistically significant three-way interaction between recovery strategy, time, and age group for any of the measures (*p*>0.05).

**Table 2:** Results of the three-way mixed ANOVA, employing the two within-subject factors recovery strategy (MMR) and time of measurement (T) and the between-subject factor age group.

Measure	Recovery strategy x time x age group interaction			Recovery strategy x time interaction			Recovery strategy x age group interaction			Time x age group interaction			Recovery strategy			Time			Age group		
	*F*	*p*	$\eta_{p}^{2}$	*F*	*p*	$\eta_{p}^{2}$	*F*	*p*	$\eta_{p}^{2}$	*F*	*p*	$\eta_{p}^{2}$	*F*	*p*	$\eta_{p}^{2}$	*F*	*p*	$\eta_{p}^{2}$	*F*	*p*	$\eta_{p}^{2}$
MVIC force in the half squat (N)	11.212	0.313	0.080	1.709	0.199	0.109	0.192	0.668	0.014	0.441	0.695	0.031	0.093	0.764	0.007	19.818	0.001	0.586	9.691	0.008	0.409
MVIC force in the leg press (N)	0.285	0.778	0.020	0.257	0.799	0.018	0.627	0.442	0.043	0.643	0.542	0.044	0.015	0.903	0.001	12.106	0.001	0.464	6.486	0.023	0.317
Resting twitch force (N)	0.753	0.509	0.051	0.707	0.533	0.048	0.724	0.409	0.049	2.655	0.077	0.159	2.517	0.135	0.152	46.316	0.001	0.768	3.762	0.073	0.212
CMJ height (cm)	0.672	0.614	0.046	1.384	0.251	0.090	0.675	0.425	0.046	1.805	0.141	0.114	2.424	0.142	0.148	53.725	0.001	0.793	35.787	0.001	0.719
Creatine kinase (U/l)	0.407	0.594	0.028	3.210	0.078	0.187	0.288	0.600	0.020	2.260	0.125	0.139	0.010	0.923	0.001	36.032	0.001	0.720	3.049	0.103	0.179
Muscle soreness (1–10)	0.281	0.889	0.020	2.956	0.028	0.174	0.039	0.846	0.003	3.792	0.008	0.213	3.367	0.088	0.194	35.950	0.001	0.720	0.018	0.895	0.001
PPC (0–6)	1.383	0.251	0.090	2.947	0.028	0.174	0.130	0.724	0.009	3.307	0.017	0.191	4.358	0.056	0.237	19.500	0.001	0.582	1.678	0.216	0.107

*MVIC, maximal voluntary isometric contraction; CMJ, countermovement jump; and PPC, perceived physical performance capability*.

The individual results revealed an inconsistent response (i.e., multivariate perspective) in most cases; thus, the response to MMR was apparently random in both young and master athletes. In addition, some participants showed a positive response to MMR in subjective markers, but not in most objective markers or *vice versa* (e.g., participants 4 and 14). Only participants 2, 8, and 10 seemed to respond more or less consistently positive to MMR, while there was no consistently negative response to MMR in any of the participants (Table 3).

**Table 3:** Individual net effect differences between a mixed-method recovery (MMR) intervention and a passive recovery (PAS) on selected markers between post-training and post-48 measures.

	Participants															
	Young athletes								Older athletes							
	1	2	3	4	5	6	7	8	9	10	11	12	13	14	15	16
Countermovement jump height (cm)	−	+	−	+	+	+	+	+	○	+	+	○	○	−	−	○
MVIC force in the half squat (N)	+	+	−	+	○	+	−	+	−	○	−	+	○	−	+	○
MVIC force in the leg press (N)	−	+	+	+	○	−	−	+	−	+	−	+	+	−	+	−
Resting twitch force (N)	+	+	−	+	−	+	−	○	○	+	○	+	+	−	−	+
Creatine kinase (U/l)	−	−	○	+	−	−	+	+	−	+	+	−	+	−	−	−
Muscle soreness (0–10)	+	+	+	−	+	−	+	+	+	+	+	+	−	+	−	○
Perceived physical performance capability (0–6)	+	+	−	−	○	+	+	+	○	+	○	−	−	+	+	○

Individual effect sizes were determined by calculating the individual differences in the changes between MMR and PAS and dividing the result by the pooled individual pretraining SD. A substantial positive (+) or negative (−) difference in the changes between MMR and PR was indicated when the effect size was greater than 0.35 (Buchheit et al., 2020). MVIC, maximal voluntary isometric contraction; ○, trivial difference in the changes between MMR and PR.

The two-way mixed ANOVA showed a statistically significant within-subject effect (*p*<0.05) of squat exercise session for mean HR, as it was higher during the exercise session performed before PR compared to the exercise session performed before MMR (Table 4). In addition, a statistically significant between-subject effect (*p*<0.05) of age group was observed for mean and peak HR and BLa concentration, which were all higher in young athletes. There was no statistically significant two-way interaction between exercise session and age group (*p*>0.05).

**Table 4:** External and internal squat training loads as well as results of the two-way mixed ANOVA, employing the within-subject factor squat exercise session and the between-subject factor age group.

Measure	Exercise session followed by MMR		Exercise session followed by PR		Exercise session x age group interaction			Exercise session			Age group		
	Young athletes	Older athletes	Young athletes	Older athletes									
	Mean±95% confidence intervals				*F*	*p*	$\eta_{p}^{2}$	*F*	*p*	$\eta_{p}^{2}$	*F*	*p*	$\eta_{p}^{2}$
Workload (kg)	6544 ± 473	6139 ± 488	6667 ± 644	6128 ± 420	0.139	0.714	0.010	0.097	0.760	0.007	0.734	0.406	0.050
Repetitions during all out set (n)	13 ± 2.8	11 ± 2.3	14 ± 3.1	10 ± 1.2	0.248	0.626	0.017	0.097	0.760	0.007	1.038	0.326	0.069
HR_mean_ (bpm)	132 ± 4.3	104 ± 6.5	134 ± 5.7	109 ± 6.8	0.153	0.702	0.011	5.275	0.038	0.274	17.861	0.001	0.561
HR_peak_ (bpm)	179 ± 3.5	151 ± 4.9	182 ± 4.8	152 ± 4.6	0.450	0.513	0.032	1.582	0.229	0.102	27.825	0.001	0.665
BLa_post_ (mmol/l)	10.8 ± 0.7	6.9 ± 0.6	10.0 ± 0.7	6.7 ± 0.6	0.327	0.577	0.023	0.862	0.369	0.058	11.791	0.004	0.457
RPE_post_ (0–10)	7.8 ± 0.9	7.0 ± 0.9	7.6 ± 0.8	7.0 ± 0.9	0.033	0.859	0.002	0.033	0.859	0.002	0.757	0.399	0.051

*MMR, mixed-method recovery; PR, passive recovery; HR, heart rate; RPE, rating of perceived exertion; and BLa, blood lactate concentration*.

## Discussion

The study aimed at investigating the effects of aging on muscle soreness and muscle damage following an intensive squat exercise protocol as well as on age-related effects of a recovery intervention in tightly controlled populations of young and master athletes regarding their absolute performance level. As an important experimental precondition, all markers of fatigue and recovery showed a significant within-subject effect of time of measurement with strikingly more pronounced changes from pre- to post-exercise (Figure 2), while CK peaked at post-24 in most individuals. Usually, the highest CK activity is found at 24 or 48h post-exercise. A mean CK increase of up to two times the baseline and up to 800U/l in some individuals can be attributed to structural damage to muscle cells (Baird et al., 2012). Therefore, it can be concluded that the applied high-intensive and eccentric overloaded exercise protocol was acutely sufficiently fatiguing and well-chosen to produce ongoing muscle soreness and muscle damage, which confirms the findings from other studies (Proske and Allen, 2005; Lavender and Nosaka, 2006; Raeder et al., 2016a; Bridgeman et al., 2017; Thomas et al., 2018).

Regarding the first hypothesis, the master athletes neither reach higher fatigue levels nor recover more slowly than the younger athletes (rejection of hypothesis 1). These findings partly contradict the current literature suggesting that, for a similar exercise stimulus, a stronger level of fatigue might be reached, and a longer recovery period might be required before master athletes return to baseline levels (Fell and Williams, 2008). A faster acute recovery is, among other factors, attributed to physiological differences between young and older participants (e.g., phosphocreatine recovery and impaired oxygen supply; Fell and Williams, 2008). Regarding long-term recovery, the common belief among athletes and coaches is that older athletes experience greater muscle damage and slower repair during and after highly intensive exercise because of minor anabolic stimulus. On the other hand, this aspect is still debated in the literature, since multiple methodological issues have to be addressed, such as gender differences, training status, training protocols, and measurements of the damage (Fell and Williams, 2008).

It must be pointed out that master athletes experienced less pronounced acute internal load and perceptual fatigue (Tables 2 and 3, and Figure 2). Similarly, the PPC showed a lower acute post-exercise decrease in the master athletes compared with the young athletes. The higher BLa concentrations in the young athletes can be attributed to the higher number of repetitions during the final all-out set when participants were encouraged to perform until their momentary muscular failure (Table 3). It appears that the master athletes started into the final set more carefully and defensively while anticipating possible injury risks and the upcoming recovery demands. Furthermore, despite a similar leg muscle mass and a comparable level of maximum dynamic strength (Table 1), the samples clearly differed in their power capacities during the vertical jump test (Figure 1). A higher amount of type II fibers in younger athletes and a correspondingly stronger glycolytic activity can be assumed. Type II fibers are usually more susceptible to disruption leading to higher BLa and CK values in young athletes (Baird et al., 2012). Therefore, a final assessment of hypotheses 1 is difficult as internal and external loads were slightly different in both age groups.

Regarding the second hypothesis, it was assumed that CWI and CC, especially when used in combination and over a longer period, could improve post-exercise recovery by ameliorating edema, decreasing secondary exercise-induced muscle damage and the sensation of muscle soreness, improving the clearance of muscle metabolites, and increasing post-exercise parasympathetic activity (Ihsan et al., 2016; Brown et al., 2017). However, on the group level and regardless of age, the current study revealed no effect of MMR on the recovery of performance or muscle contractile markers of fatigue and recovery (rejection of hypothesis 2). Additionally, MMR did not significantly improve the recovery of CK activity, although reductions in muscle soreness and improvements in PPC were evident after MMR. Such a finding is common, likely due to the analgesia and placebo effect attributable to CWI and CC (Leeder et al., 2012; Poppendieck et al., 2013; Ihsan et al., 2016; Brown et al., 2017).

Regardless of age, the group-level results of the current study are both consistent and in contrast to the overall literature findings examining CWI or CC as recovery tools, with applied research showing both improved and unchanged or even impaired recovery of fatigue markers following CWI and CC (Leeder et al., 2012; Poppendieck et al., 2013; Hill et al., 2014; Machado et al., 2016; Marqués-Jiménez et al., 2016; Brown et al., 2017). The inconsistent findings within the available studies can be explained by the different CWI and/or CC protocols used as well as the frequently reported extensive interindividual variations even in response to highly standardized training and/or recovery interventions (Pickering and Kiely, 2017; Wiewelhove et al., 2021). A high interindividual variability in the observed response to recovery interventions is supported by the current study. Only three participants (i.e., participants 2, 8, and 10) did likely benefit from MMR. All other participants showed inconsistent/diffuse responses, while a positive effect of MMR on the recovery of performance tended to be more frequently observed in the younger athletes. The inconsistent responses could be explained by a possible inability of the MMR protocol to accelerate recovery, at least in terms of muscle performance and CK activity, as well as the complexity of the kinematics of exercise-induced fatigue, which may cause different effects within individual body systems. Genetic influences make an important contribution to these variations, while factors, such as sleep, psychological stress, habitual physical activity, and dietary intake, may also play important roles (Mann et al., 2014). Only dietary intake was standardized and controlled during the present study. Additionally, the amount of data was rather small. Thus, the effectiveness of MMR should be interpreted with great caution.

It is also assumed that the effectiveness of a recovery intervention may differ more depending on the individual preferences and beliefs concerning the intervention rather than on age (Roelands and Hurst, 2020). For example, several participants from both age groups (i.e., participants 1, 2, 7, 8, 10, 14, and 16) showed a consistent positive response to MMR in subjective markers, but MMR likely only had a consistent positive impact on performance in participant 8, while the performance of participant 14 was likely to have benefited from PR. Conversely, participant 4 showed a positive response to MMR in MVIC force, resting twitch force, CMJ height, and serum concentration of CK, but had a negative response to MMR in both subjective markers. Therefore, the use of recovery methods post-exercise in general and of MMR in particular should be individualized regardless of age. However, considering only individual perceptions may be misleading for the restoration of performance and/or muscle function.

Furthermore, it is discussed in the literature whether a long-term application of recovery interventions might attenuate training-specific adaptations in long-term training intervention and how to balance possible deteriorations in training adaptations with the possible beneficial short-term recovery effects. For example, Fröhlich et al. (2014), Roberts et al. (2015), and Poppendieck et al. (2020) showed that the regular use of CWI after strength training sessions reduced long-term gains in muscle mass and strength. However, the effects were rather small and therefore only of practical relevance for a few elite athletes. In this study, MMR had not physiological effects on post-exercise recovery at the group level. Thus, possible negative effects of a long-term application of MMR on adaptations are most likely rather minor and negligible in most athletes.

Finally, some limitations of the study design must be considered which are (1) the small number and heterogeneity of selected young and elderly athletes and (2) the insufficient adjustment of internal and external loads. Regarding the sample size, we strictly followed the predefined inclusion criteria for both age groups, which were difficult to meet by the master athletes. More than fifty applicants had to be pre-tested to identify eight individuals reaching a minimum half squat 1RM of at least 120% of the individual body weight which was necessary to match the dynamic strength performance of both age groups and corresponds to an advanced resistance training status in older athletes (Santos Junior et al., 2021). Nevertheless, despite a comparable level of maximum dynamic strength and a similar body composition, the samples clearly differed in their power capacities. Noticeable in this regard were the significantly lower CMJ performance and twitch force (Figure 2) as well as the lower basic testosterone release in the older athletes (Table 1). It can be concluded that a complete performance matching was not realized; however, it was implemented as well as possible at least for the 1RM, which was defined as our primary inclusion criterion.

Differences in the acute internal and external loads of the exercise program can be attributed to the higher number of repetitions during the final set as well as to a higher amount of type II fibers and a correspondingly stronger glycolytic activity in the younger athletes. Nevertheless, since no significant within-subject effect between the two cross-over parts was found for any of the fatigue and recovery markers, it appears that a sufficiently standardized and replicable protocol was prescribed.

## Conclusion

At the group level, master athletes neither reach higher fatigue levels nor recover more slowly than the younger athletes. Age-related differences were only found within younger athletes reaching a higher post-exercise fatigue regarding muscle soreness and PPC. Furthermore, the results indicate that a mixed-method recovery intervention (MMR) after resistance exercise does not contribute to a faster recovery of physical performance, neuromuscular function, or muscle damage, but promotes recovery of perceptual measures regardless of age. However, some participants were more likely to benefit from MMR also in terms of functional and physiological measures. Therefore, the use of MMR should be individualized. As limitations, it has to be pointed out that our sample size was rather small and that a complete matching of population characteristics and exercise intensity was difficult under the ecological valid conditions of this study. Both may have affected the outcomes. We therefore recommend further studies under more controlled experimental conditions.

## Data Availability Statement

The datasets presented in this study can be found in online repositories. The original dataset is available *via* the Open Science Framework at: https://osf.io/udtpz/?view_only=2964e65f6cc146c893852a13ac71c286.

## Ethics Statement

The studies involving human participants were reviewed and approved by the Local Ethics Committee of the Faculty of Sport Science of the Ruhr University Bochum. The patients/participants provided their written informed consent to participate in this study.

## Author Contributions

JS, AF, and TW planned and designed the experimental design and prepared the manuscript. JS, AF, NV, and JW performed the experiments. JS and TW analyzed the data. AF, MK, MP, and OB contributed to materials and analysis tools. MK, FB, MP, NV, JW, and OB edited the manuscript. All authors read and approved the submitted version.

## Funding

The current study was funded by the German Federal Institute of Sport Science (http://www.bisp.de). The research was realized in the project REGman–Optimization of Training and Competition: Management of Regeneration in Elite Sports (IIA1-081901/17–20). Funding were received by MK, MP, and AF. We further acknowledge support by the DFG Open Access Publication Funds of the Ruhr University Bochum. The funders had no role in study design, data collection and analysis, decision to publish, or preparation of the manuscript.

## Conflict of Interest

The authors declare that the research was conducted in the absence of any commercial or financial relationships that could be construed as a potential conflict of interest.

## Publisher’s Note

All claims expressed in this article are solely those of the authors and do not necessarily represent those of their affiliated organizations, or those of the publisher, the editors and the reviewers. Any product that may be evaluated in this article, or claim that may be made by its manufacturer, is not guaranteed or endorsed by the publisher.

## Footnotes

^1^ https://www.myfitnesspal.com
